# Cytokine dynamics and quality of life: unraveling the impact of cell-free and concentrated ascites reinfusion therapy in ovarian cancer patients

**DOI:** 10.1007/s10147-024-02682-1

**Published:** 2025-01-04

**Authors:** Rikako Ito, Masahiro Kagabu, Sho Sato, Eriko Takatori, Yoshitaka Kaido, Takayuki Nagasawa, Tadahiro Shoji, Takashi Hirayama, Yasuhisa Terao, Tsukasa Baba

**Affiliations:** 1 Department of Obstetrics and Gynecology, Iwate Medical University School of Medicine, 2-1-1, Idaidori, Yahaba, Iwate 028-3695 Japan; 2 Department of Obstetrics and Gynecology, Faculty of Medicine, Juntendo University, 3-1-3 Hongo, Bunkyo-ku, Tokyo, Japan

**Keywords:** Cell-free and concentrated ascites reinfusion therapy, Cytokine, Ovarian cancer, Interleukin-6, Malignancy, Quality of life

## Abstract

**Background:**

The quality of life (QOL) of ovarian cancer patients is often impaired by refractory ascites. Cell-free and concentrated ascites reinfusion therapy (CART) is a palliative treatment for refractory ascites, but adverse events, such as fever, are problematic. Several cytokines have been suggested to be responsible for the adverse events, but they have not been investigated in detail. Thus, we comprehensively analyzed cytokines in ascites fluid (AF) and serum before and after CART to determine the influence of cytokines on the safety and efficacy of CART.

**Methods:**

Thirteen ovarian cancer patients with refractory malignant ascites who underwent CART were enrolled. We comprehensively analyzed 27 cytokines in AF and serum before and after CART. Simultaneously, vital measurements, blood tests, adverse event recordings, and QOL assessments were performed to examine the relationships between the cytokines in AF and serum.

**Results:**

Interleukin (IL)-5, IL-6, IL-10, and monocyte chemoattractant protein-1 levels were increased in the concentrated AF and in the serum immediately after reinfusion, but they decreased after 24 h. Body temperature also increased immediately after reinfusion, and decreased after 24 h. The CRP level at 24 h after reinfusion was increased, and was positively correlated with the IL-6 level. A QOL assessment using the Cancer Fatigue Scale revealed significantly lower scores after CART.

**Conclusions:**

The results indicate that the cytokine-induced fever and increased inflammatory response after CART were temporary, and that CART is safe. Additionally, QOL improved after CART. Thus, CART appears safe and effective for treating patients with refractory cancerous ascites.

**Supplementary Information:**

The online version contains supplementary material available at 10.1007/s10147-024-02682-1.

## Introduction

According to the 2022 GLOBOCAN Global Cancer Women’s Cancer Data, ovarian cancer is the eighth most common cancer among women worldwide, and with a 5-year survival rate of 30–50%, it has the highest mortality rate among gynecological tumors [1]. In general, the prognosis for ovarian cancer is poor, because the disease is often diagnosed at an advanced stage. Many patients with ovarian, fallopian tube, and/or peritoneal cancer (hereafter called “ovarian cancer patients”) develop refractory ascites as their disease progresses. A significant increase in ascites causes symptoms, such as abdominal distention, anorexia, and dyspnea, leading to poor quality of life (QOL) [2, 3]. Therefore, to maintain QOL for patients with advanced ovarian cancer, it is important to control refractory ascites. Most patients with advanced ovarian cancer eventually become resistant to chemotherapy and present with refractory ascites, often requiring repeated abdominal paracentesis to relieve symptoms [4]. More recently, cell-free and concentrated ascites reinfusion therapy (CART) has gained recognition as a treatment for patients with refractory ascites. In CART, ascites fluid (AF) is collected and filtered through a membrane to remove bacteria, cancer cells, and blood cell components, and then, the remaining albumin and other protein components are concentrated in a concentrator membrane and returned to the patient intravenously. CART is more useful than simple ascites punctures, because it improves patients’ QOL by improving their general health and nutritional status, and because the use of recovered autologous protein reduces the need for albumin products [5, 6]. CART has also been used as an early palliative treatment for malignant ascites due to gynecological cancer, and has been reported to relieve symptoms and improve patients’ QOL [7]. Several protocols for CART have been developed to optimize its efficacy and safety. In some instances, heparin is added to the ascites to prevent fibrin formation during the procedure, while premedication with hydrocortisone is occasionally administered to mitigate the risk of fever [8]. KM-CART, an enhanced version of the conventional CART, has been specifically designed to address malignant ascites. This system incorporates a membrane-cleaning function that effectively resolves filtration membrane blockages, allowing for the processing of larger volumes of ascites. KM-CART is particularly utilized in cases of advanced ovarian cancer characterized by significant ascites accumulation, where it has been reported to alleviate subjective symptoms such as abdominal distension [9, 10].

In previous studies, CART helped maintain the serum albumin levels and prolong the time before ascites reaccumulate when compared to simple ascites punctures [6]. However, the biological effects of the components in AF after CART have yet to be sufficiently investigated. A relationship between CART and AF components has been reported, since inflammatory cytokines, such as interleukin (IL)-1β, IL-6, IL-8, tumor necrosis factor (TNF)-α, and IL-10, were found in AF; in addition, no association between these cytokines and adverse events, such as fever, was seen [11]. It has been established that cytokines are involved in the mechanism of fever, and that IL-1, tumor necrosis factor (TNF), and IL-6 are important mediators of fever induction. Cytokines are thought to act in the brain to enhance the synthesis of cyclooxygenase 2 (COX2), an enzyme that produces prostaglandin E2 (PGE2), causing fever [12]. However, there have been no studies to date that have investigated the cytokines in serum before and after CART. There have also been no reports of cytokine release syndrome after CART.

In the present study, we investigated the effects of cytokines on the body when CART is performed. Additionally, we examined the impact of CART on QOL in ovarian cancer patients.

## Methods

This was a prospective cohort study analyzing refractory AF components and the impact of CART in ovarian, fallopian tube, and/or peritoneal cancer patients with refractory ascites. We examined 13 patients with ovarian, fallopian tube, and/or peritoneal cancer with refractory ascites who underwent CART between December 1, 2017, and March 31, 2020.

This study was performed in accordance with the tenets of the Declaration of Helsinki and the ethical guidelines for epidemiologic research of the Ministry of Health, Labour and Welfare of Japan. The study was approved by the Ethics Committee of Iwate Medical University School of Medicine (MH2021-065), and was registered in the University Hospital Medical Information Network Clinical Trial Registry (UMIN-CTR) under UMIN000034893. Patients provided written informed consent before enrollment into the study.

### Participants

Patients were enrolled at Juntendo University Hospital and Iwate Medical University Hospital. The eligibility criteria were: (1) ovarian cancer patients with refractory malignant ascites, (2) patients who could undergo abdominal paracentesis, (3) patients who provided written informed consent, and (4) patients who were at least 20 years of age and less than 80 years of age; all criteria had to be met for inclusion. The exclusion criteria were: (1) patients who have fever and suspected endotoxin detection, (2) severely immunocompromised patients requiring bone marrow transplantation, (3) patients who have severe cardiac, renal, or hepatic insufficiency, (4) patients who have bacterial peritonitis, (5) patients who have received bevacizumab within the previous 6 weeks, (6) patients who have undergone CART within the previous 4 weeks, (7) patients who are participating in other clinical trials or clinical studies, and (8) patients who are deemed inappropriate for inclusion by the treating physician. Furthermore, no increase in concomitant medications, no use of new medications, and no use of steroids for fever prophylaxis were permitted until the end of the study.

### Procedure for CART

After enrollment, patients received CART when an ascites puncture was clinically necessary (primarily due to the worsening of symptoms from AF accumulation). CART was performed using standard methods [13]. Under local anesthesia, AF was collected by gravity into a sterilized collection bag. The drainage volume for each patient was determined by the physician who was primarily responsible for the patient. The AF was filtered using AHF-MO (Asahi Kasei Medical Corporation, Tokyo, Japan), and concentrated using AHF-UP (Asahi Kasei Medical Corporation). The infusion rate was determined by the attending physician according to the manufacturer’s protocol.

### Data collection and statistical analysis

Laboratory data were analyzed before and after CART. The serum cytokine levels were also measured at the same timepoints. The total protein, albumin, and cytokines in AF before and after CART were also analyzed (Table 1). The serum and AF samples were kept frozen at − 80 °C until used for this study. We quantified 27 cytokines using the Bio-Plex 200 multiplex cytokine array system (Bio-Rad Laboratories, CA, USA) according to the manufacturer’s instructions. Cytokine levels were measured using the Bio-Plex Pro Human Cytokine GI Assay Kit 27-Plex Panel (Bio-Rad Laboratories), which includes 27 cytokines and chemokines (eotaxin, G-CSF, GM-CSF, IFN-γ, IL-1β, IL-1ra, IL-2, IL-4, IL-5, IL-6, IL-7, IL-8, IL-9, IL-10, IL-12p70, IL-13, IL-15, IL-17, IP-10, MCP-1, MIP1-α, MIP-1β, PDGF-BB, RANTES, TNF-α, and VEGF). Bio-Plex Manager software version 5.0 (Bio-Rad Laboratories) was used for cytokine data acquisition and analysis. Body temperature (BT) was measured the morning of the day of abdominal paracentesis, before reinfusion, immediately after reinfusion, and the day after reinfusion (morning, noon, and evening). QOL was measured using the Cancer Fatigue Scale (CFS). The CFS consists of three categories: physical fatigue, mental fatigue, and cognitive fatigue. There are 15 questions and it is a self-administered questionnaire that is scored on a scale of 1–5. A score of 0 indicates no fatigue at all, and higher scores indicate more severe fatigue. The maximum possible score is 28 for physical fatigue, 16 for mental fatigue, 16 for cognitive fatigue, and 60 for total fatigue. The CFS can be used to assess fatigue in cancer patients in a simple and objective way [14]. The evaluations were conducted before AF collection and the day after reinfusion (within 24 h).

**Table 1:**
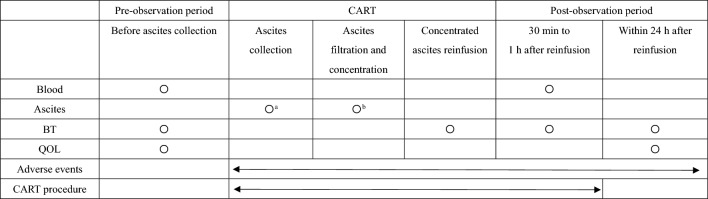
The research protocol

*BT* body temperature, *QOL* quality of life, *CART* cell-free and concentrated ascites reinfusion therapy

^a^The collected ascites was measured

^b^The ascites after filtration and concentration was measured

The cytokine data are presented as the mean ± standard deviation; other data are presented as the median (range) or percentage (%). Statistical analyses were performed using R 4.4.0 (R Core Team 2019, Vienna, Austria). Comparison of the values before and after CART in each patient was performed using Student’s t test. Single regression analysis was performed to evaluate the association between cytokines and the BT and blood laboratory data. For all analyses, a *p* < 0.05 was considered to indicate a statistically significant difference.

## Results

### Patient characteristics

The patient characteristics are shown in Table 2. The median age was 67 years. The histological types were serous carcinoma in 6 patients, clear cell carcinoma in 2 patients, carcinosarcoma in 2 patients, and endometrial carcinoma in 1 patient. The stages based on the International Federation of Gynecology and Obstetrics (FIGO) 2014 classification were stage IIIB in 2 cases, stage IIIC in 8 cases, and stage IVB in 1 case (Table 2).

**Table 2:** Patient characteristics

	Median (range)
Age (years)	67 (50–78)
Height (cm)	154.9 (142.5–161.2)
Body weight (kg)	51.4 (34.5–70.0)
Body mass index (kg/m^2^)	19.8 (14.9–29.5)
FIGO 2014 stage	*N* (%)
IIIB	2 (18.2)
IIIC	8 (72.7)
IVB	1 (9.09)
Pathologic tissue	*N* (%)
Serous carcinoma	6 (54.5)
Endometrial carcinoma	1 (9.09)
Clear cell carcinoma	2 (18.2)
Carcinosarcoma	2 (18.2)

### Analysis of the blood and AF

**Fig. 1:**
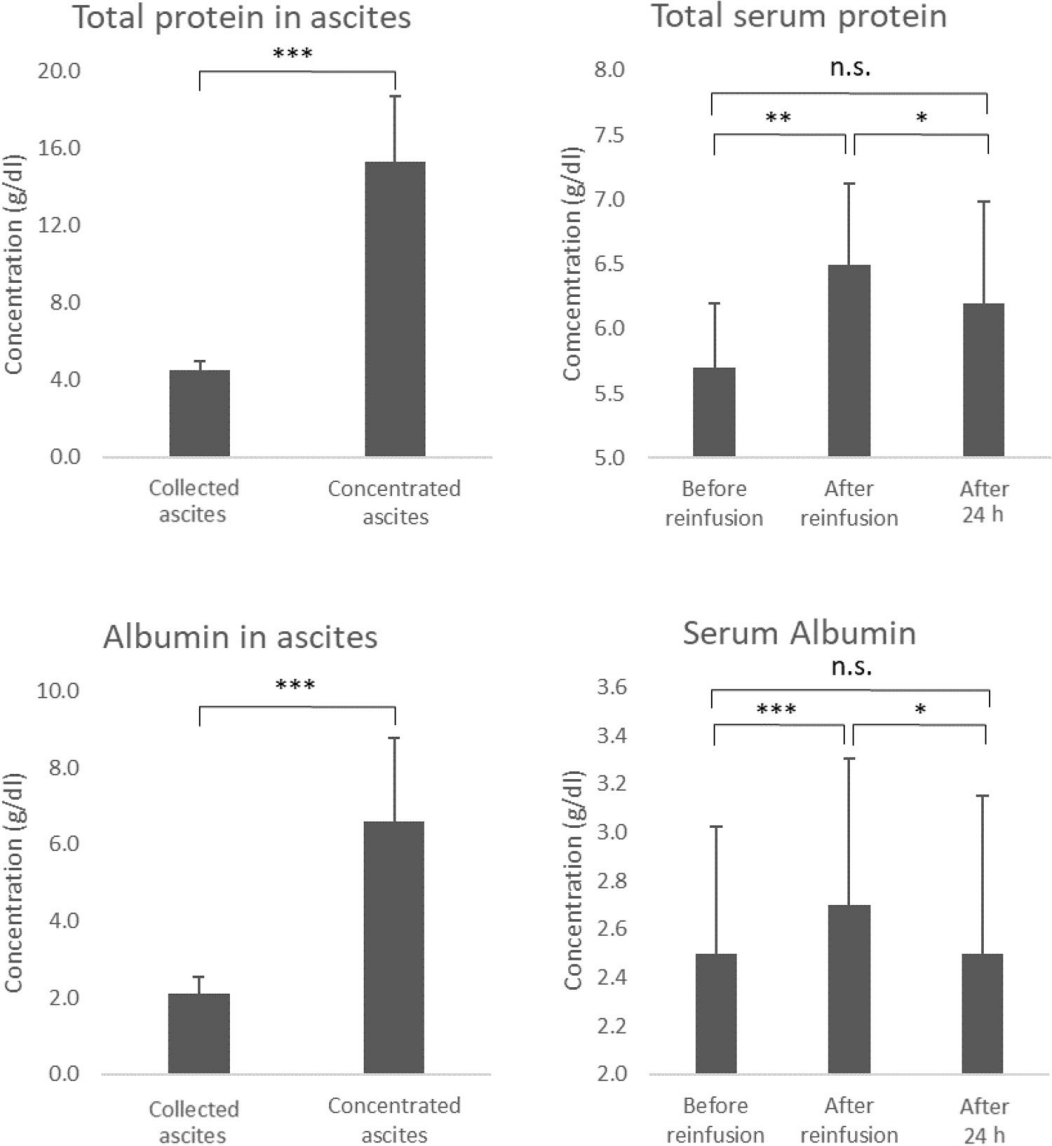
Total protein and albumin levels in the drained and concentrated ascites fluids. Total protein and albumin levels in the serum before and after CART. *p* values were determined using Student’s *t* test. *p < 0.05, **p < 0.01, ***p < 0.001, *n.s.* not significant

The median drained AF volume was 3880 (range 2390–6180) ml, and the median volume of the AF after filtration and concentration was 577 (285–1774) ml. The median total protein concentration in the drained AF was 4.5 (3.5–5.0) g/dl, and it increased to 15.3 (11.1–21.0) g/dl in the concentrated AF. The albumin concentration in the drained AF was 2.1 (1.3–2.6) g/dl, and it increased to 6.6 (5.9–12.0) g/dl in the concentrated AF. The total serum protein concentration was 5.7 (4.8–6.7) before AF drainage, 6.5 (5.1–7.2) g/dl immediately after concentrated AF reinfusion, and 6.2 (4.5–6.9) g/dl the day after reinfusion. The serum albumin level was 2.5 (1.5–3.2) g/dl before AF drainage, 2.7 (1.7–3.8) g/dl immediately after reinfusion, and 2.5 (1.4–3.7) g/dl the day after reinfusion. Taken together, both the total protein and albumin concentrations increased in the AF after filtration and concentration. Although the serum total protein and albumin concentrations were increased immediately after reinfusion, they were decreased the day after, and were only slightly higher than before AF drainage (Fig. 1, Table 3).

**Table 3:** The ascites fluid volumes and amounts of total protein and albumin in the serum and ascites

	Median (range)
Drained ascites (ml)	3880 (2390–6180)
Concentrated ascites (ml)	577 (285–1774)
Total protein in drained ascites (g/dl)	4.5 (3.5–5.0)
Total protein in concentrated ascites (g/dl)	15.3 (11.1–21.0)
Albumin in drained ascites (g/dl)	2.1 (1.3–2.6)
Albumin in concentrated ascites (g/dl)	6.6 (5.9–12.0)
Total serum protein before reinfusion (g/dl)	5.7 (4.8–6.7)
Total serum protein after reinfusion (g/dl)	6.5 (5.1–7.2)
Total serum protein after 24 h (g/dl)	6.2 (4.5–6.9)
Serum albumin before reinfusion (g/dl)	2.5 (1.5–3.2)
Serum albumin after reinfusion (g/dl)	2.7 (1.7–3.8)
Serum albumin after 24 h (g/dl)	2.5 (1.4–3.7)

**Fig. 2:**
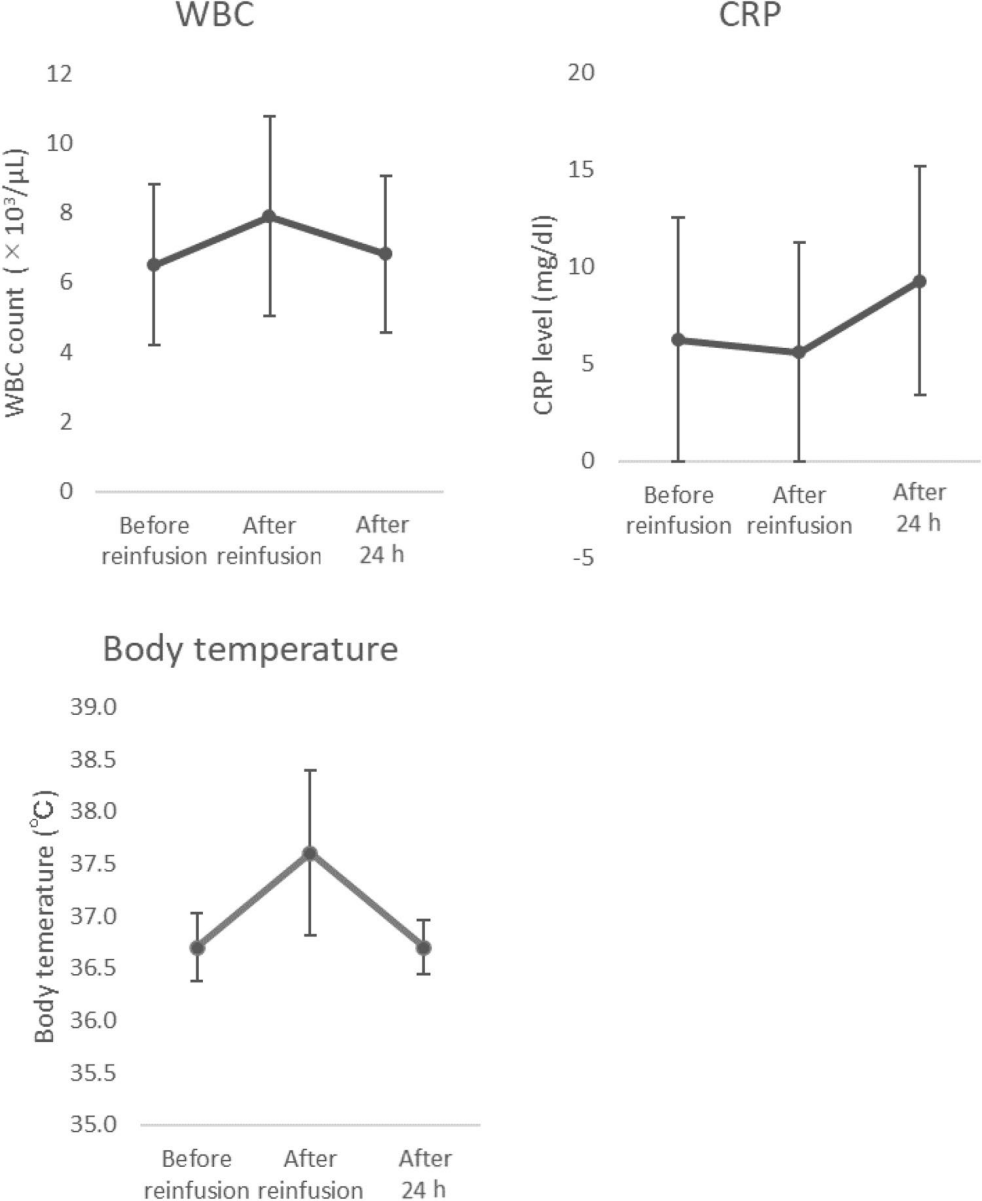
The WBC count, CRP level, and body temperature before and after CART. *WBC* white blood cell, *CRP* C-reactive protein

The median white blood cell (WBC) count was 6.5 × 10^3^ (3.0 × 10^3^–10.5 × 10^3^) cells/μl before AF drainage, 7.9 × 10^3^ (2.9 × 10^3^ to 112.6 × 10^3^) cells/μl immediately after reinfusion, and 6.8 × 10^3^ (3.1 × 10^3^–11.6 × 10^3^) cells/μl the day after reinfusion. The median CRP level was 6.26 (1.18–19.2) mg/dl before AF drainage, 5.62 (0.78 to 17.83) mg/dl immediately after reinfusion, and 9.28 (1.08–22.3) mg/dl the day after reinfusion (Fig. 2).

**Fig. 3:**
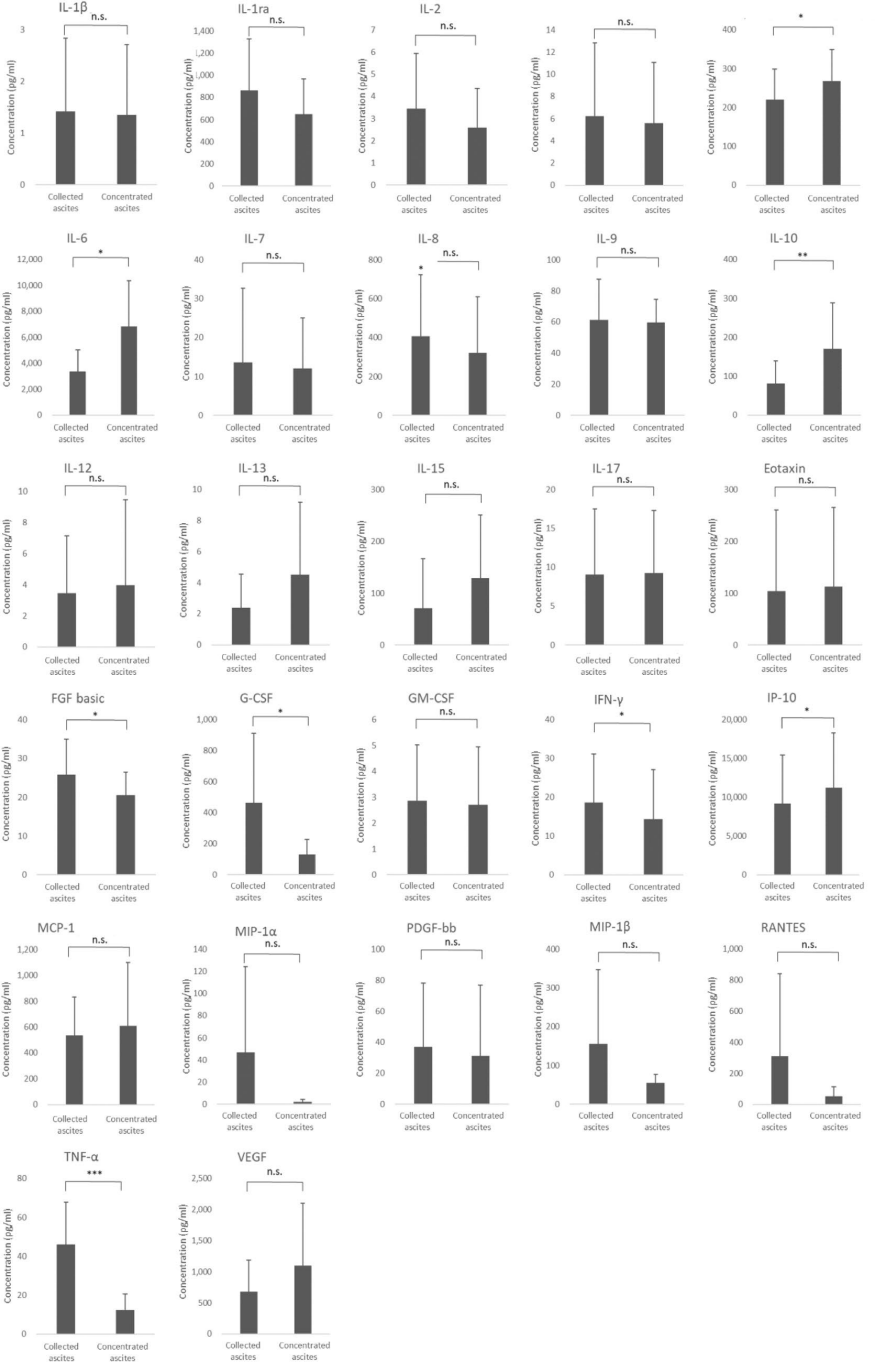
Concentrations of the 27 cytokines in the collected and concentrated ascites. *p* values were determined using Student’s *t* test. *p < 0.05, **p < 0.01, ***p < 0.001, *n.s.* not significant

We investigated 27 cytokines/chemokines in the AF after drainage, and after filtration and concentration. We found that the IL-5, IL-6, IL-10, and IP-10 levels were significantly increased after filtration and concentration. In contrast, the FGF-basic, G-CSF, IFN-γ, and TNF-α levels were significantly decreased after filtration and concentration (Fig. 3).

**Fig. 4:**
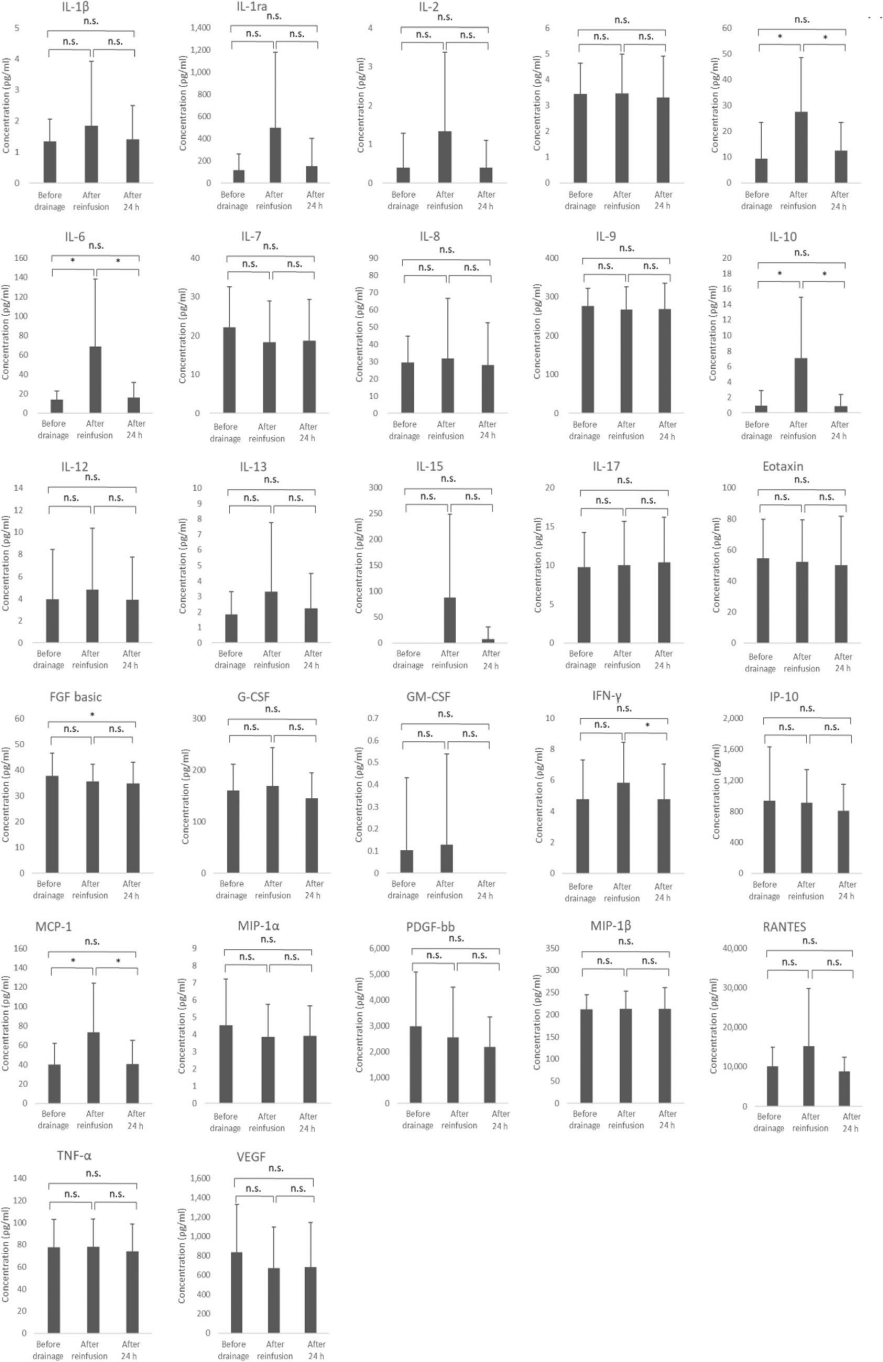
Concentrations of the 27 cytokines in blood before and after CART. *p* values were determined using Student’s *t* test. *p < 0.05, **p < 0.01, ***p < 0.001, *n.s.* not significant

The serum IL-5, IL-6, IL-10, and MCP-1 levels increased significantly immediately after reinfusion, and were significantly decreased 24 h later. However, there was no significant difference in any of the serum cytokine levels before AF drainage and 24 h after reinfusion (Fig. 4). Of note, the IL-5, IL-6, and IL-10 levels were increased in the concentrated AF, and also in the serum after reinfusion. Clear cell carcinomas have been reported to oversecrete IL-6 more than other histologic types [15]. In the present study, no significant differences were found in serum IL-6, IL-10, MCP-1, WBC, and CRP between clear cell carcinoma and other histologic types.

### Adverse events and QOL

The median BT was 36.7 °C (36.3–37.5 °C) before AF drainage, and it increased to 37.6 °C (36.3–38.6 °C) immediately after reinfusion of ascites, and then decreased to 36.7 °C (36.2–37.1 °C) the day after. When compared to before AF drainage (before CART), the mean BT increased by 0.57 °C ± 0.67 °C immediately after reinfusion, and decreased by 0.10 °C ± 0.40 °C the day after reinfusion. No adverse events, such as increased blood pressure, decreased blood pressure, infection, or decreased renal function, were observed in the patients. The median CFS score was 33 (9–43) before AF drainage, and it significantly decreased to 23.5 (6–33) the day after reinfusion (Table 4).

**Table 4:** QOL assessment with the CFS

	Median (range)
CFS score before CART	33 (9–43)
CFS score after CART	23.5 (6–33)
*p* value	0.000536

*p* values were determined by Student’s *t* test

### Correlations between BT, WBC, CRP, and IL-6

**Fig. 5:**
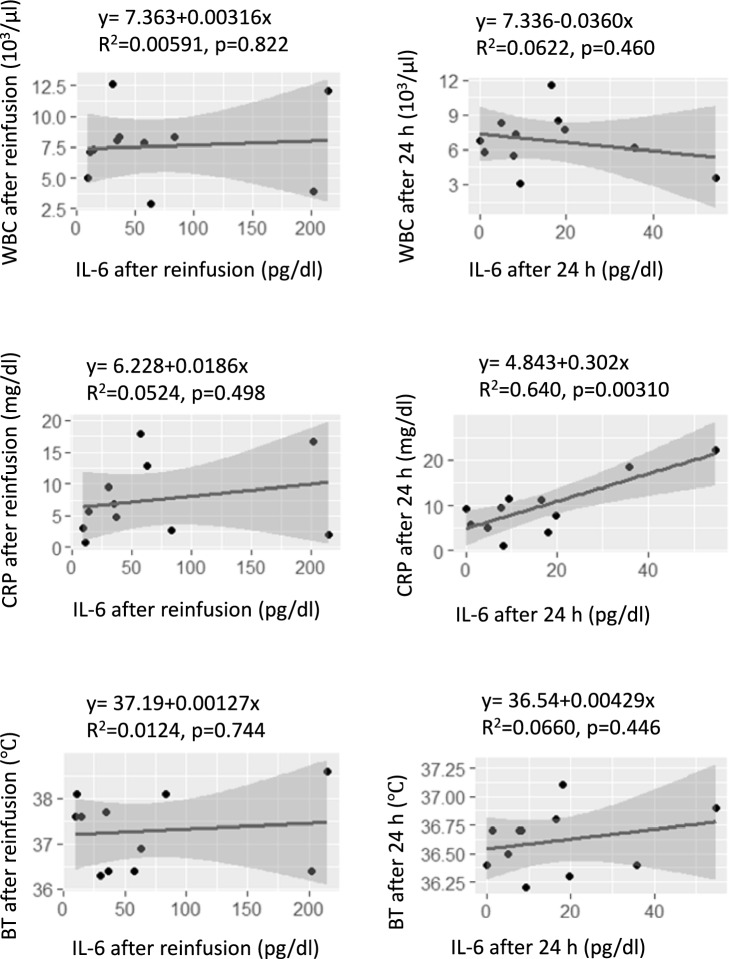
Scatter diagram showing the relationships between the WBC count, BT, and CRP and IL-6 levels in the patients after reinfusion and 24 h later. *WBC* white blood cell, *CRP* C-reactive protein, *BT* body temperature

We analyzed the correlations between the BT, WBC count, and CRP level with respect to the elevated IL-5, IL-6, and IL-10 levels that were observed immediately after reinfusion. We found that only IL-6 correlated with the BT, WBC count, and CRP level. The results from a single regression analysis of the BT, WBC count, and CRP and IL-6 levels showed that the CRP level at 24 h after reinfusion was positively correlated with the IL-6 level (*p* = 0.00310). In addition, we found that the BT (*p* = 0.744), WBC count (*p* = 0.822), and CRP level (*p* = 0.498) immediately after reinfusion, and the BT (*p* = 0.446) the day after reinfusion tended to be increased with increasing IL-6 (Fig. 5).

## Discussion

CART is an established treatment for refractory ascites, but no detailed information on biological parameters before and after CART has been published to date. Our study revealed three major points. First, the levels of four of the 27 examined cytokines, *i.e.,* IL-5, IL-6, IL-10, and MCP-1, were elevated in the concentrated AF, and their levels were also increased in blood immediately after reinfusion of concentrated ascites reduction, but decreased 24 h after reinfusion. Furthermore, among the cytokines in the concentrated AF, FGF-basic, G-CSF, IFN-γ, and TNF-α were present at lower levels in the concentrated AF than in the original AF. Second, the BT and WBC count increased immediately after reinfusion, but decreased after 24 h, unlike the CRP level, which increased 24 h after reinfusion. Moreover, a positive correlation was found between CRP and IL-6 at 24 h after reinfusion. Third, in the QOL assessment, we found that the CFS score was significantly lower the day after reinfusion than before AF collection, indicating that QOL improved after CART. These three points are further discussed below.

First, regarding cytokines in AF, the prognostic impact of inflammatory cytokines in patients with malignant tumors has been investigated in a study that reported the presence of IL-1β, IL-6, IL-8, IL-12, TNF-α, and IL-10 in AF. The presence of IL-10 was associated with a prolonged life expectancy after CART. In contrast, there was no significant correlation between the other cytokines and adverse events, such as fever, and the WBC count and CRP level, which are indicators of an inflammatory response [11]. In the present study, we similarly detected the presence of IL-1β, IL-6, IL-8, TNF-α, and IL-10 in AF; the IL-6 values were lower, the TNF-α and IL-10 values were slightly higher, and the IL-1β and IL-8 values were comparable to the values reported in the previous studies. In addition, we found that the levels of FGF-basic, G-CSF, IFN-γ, and TNF-α were lower in the concentrated AF than in the original AF. G-CSF, IFN-γ, and TNF-α are cytokines that play a role in enhancing inflammation. They are filtered out and removed by the AF filtration unit (AHF-MO). Rather than suppressing inflammation, the removal of these cytokines in CART does not cause inflammation. In particular, TNF-α is a cytokine that is secreted in the early stages of inflammation, and it has a half-life of less than 20 min; despite its short half-life, it is believed to induce the secretion of various inflammatory and anti-inflammatory cytokines, such as IL-6, IL-8, IFN-γ, and IL-10 [16]. The IL-5, IL-6, IL-10, and MCP-1 levels in blood were increased immediately after reinfusion, but were decreased 24 h later, likely because these cytokines have short half-lives ranging from a few minutes to around 20 h [17]. These results indicate that the levels of the cytokines induced by TNF-α that were found in the concentrated AF would have also decreased naturally in the body, suggesting that even though the reinfusion of concentrated AF returns the inflammatory cytokines into the body, the effect of the cytokines is transient. Thus, suggesting that CART is safe.

Regarding the second point, the BT and WBC count increased immediately after reinfusion, but decreased 24 h later, and that the CRP level increased 24 h after reinfusion. In addition, we observed a positive correlation between the CRP and IL-6 levels at 24 h after reinfusion. Previous studies found no correlation between cytokines and adverse events, such as an elevated BT, WBC count, and CRP level, which are associated with inflammatory responses [11]. In our study, the results of the single regression analysis showed that the CRP level at 24 h after reinfusion was positively correlated with the IL-6 level. Moreover, the BT, WBC count, and CRP level immediately after reinfusion, and the BT and CRP level on the day after reinfusion tended to be increased with increasing IL-6 levels. IL-6 is a very important cytokine in the early host response to infection, and an increase in IL-6 precedes an increase in CRP. IL-6 has a very short half-life, becoming undetectable within 24 h in most infected patients. CRP is synthesized in the liver within 6–8 h in response to inflammation, peaking at 24–48 h, and then decreasing with time as the inflammation subsides [18]. Previous studies found that the CRP level was increased 3 h after IL-6 administration, and was further increased at 16 h. It has also been reported that the number of neutrophils was increased by IL-6 administration, peaking at 2 h after administration, and returning to the pre-administration level by the next day [19]. In the present study, the CRP level did not change immediately after reinfusion, but was increased 24 h later; in contrast, the WBC count increased immediately after reinfusion, and was decreased after 24 h. This suggested that the WBC count increased immediately after reinfusion and the CRP level increased 24 h later due to the immediate increase in IL-6 that occurred after reinfusion. Thus, the inflammation caused by CART is considered to be temporary, as the WBC count and IL-6 level peaked out at 24 h after reinfusion. In addition, fever was observed immediately after reinfusion, but it resolved 24 h later. We consider that the fever was induced by inflammatory cytokines, since the blood levels of inflammatory cytokines, such as IL-6, increased immediately after reinfusion. The fever encountered in the present study was a Grade 1 (mild) adverse event according to the Common Terminology Criteria for Adverse Events (CTCAE) Version 5.0. Thus, our results indicate that CART can be safely performed with no adverse events other than fever, which can be managed with non-steroidal anti-inflammatory drugs and acetaminophen.

As for the third point, the CFS score was significantly lower on the day after reinfusion than before AF collection, indicating that QOL was improved by CART. There is no standardized protocol for evaluating QOL improvement with CART. Previous retrospective studies have reported that CART for cancer patients improved their QOL, with significant reductions in the circumferences of the abdomen and both thighs, and improved appetite [20]. In the previous systematic reviews, a numerical rating scale system (NRS) was used to evaluate symptom relief after CART. Abdominal distention, dyspnea, and fatigue were reduced by 6.0 (95% CI 5.59–6.51), 2.66 (95% CI 2.05–3.28), and 2.64 (95% CI 1.86–3.42) points, respectively, using a 0–10 numerical rating scale system. Overall, 17% (95% CI 0.03–0.31%) of patients reported improved performance status after CART [7]. In the previous studies, the NRS was used to separately assess abdominal distension, dyspnea, and fatigue. As a result, it was difficult to comprehensively and multidimensionally assess the QOL of individual patients. The present study, we used CFS as a scale to comprehensively and multidimensionally assess changes in QOL. The CFS assesses physical fatigue, mental fatigue, and cognitive fatigue, and can be used to quantify and objectively evaluate a patient’s sense of fatigue. In the present study, CART significantly reduced the CFS score, indicating that CART is effective in improving the cancer patients’ QOL. In the future, in evaluating the improvement in QOL provided by CART, it will be necessary to create a protocol for administering the CFS before treatment, the day after treatment, and 1 week after treatment.

There are two limitations to this study. The first is the small number of eligible patients that were analyzed. There were 13 patients who had ovarian cancer with refractory malignant ascites and underwent CART, and only 11 patients were analyzed as data were insufficient for two cases; this small number may have increased the susceptibility to random errors. The second is that the assessment after CART was only performed at 24 h. As a prospective study, it was not possible to predict changes in cytokines and CRP after 24 h before the study. In addition, the evaluation was only conducted at 24 h in this study, as the increased number of blood samples would have increased patient invasiveness. Therefore, only short-term effects are apparent. Blood tests after 24 h would allow further investigation of the impact of CART on inflammation. Future evaluation at 48 and 72 h is an issue. Continuous QOL assessment on a weekly basis, taking into account the time until reaccumulation of ascites, would further validate the effectiveness of CART. A prospective randomized-controlled phase II trial comparing ascites puncture cases with CART cases is currently underway (the JGOG9006 trial). Although the cytokine levels in AF are not being monitored, a QOL study will be conducted. We are looking forward to the results of this study.

In conclusion, the reinfusion of concentrated AF in CART decreased the levels of inflammatory cytokines after 24 h, even though the cytokines in the AF were returned back into the body. Furthermore, the CFS score was significantly lower the day after reinfusion when compared to before AF collection, and QOL improved after CART. As this study demonstrated the safety and benefits of CART, we hope that CART will be used for treating patients with refractory cancerous ascites in the future.

## Supplementary Information

Below is the link to the electronic supplementary material.

Supplementary file1 (PPTX 150 kb)

Supplementary file2 (DOCX 16 kb)

Supplementary file3 (DOCX 17 kb)

Supplementary file4 (DOCX 15 kb)

## Data Availability

All data generated and analyzed in this study and that support the findings of this study are included within this article and its supplementary information files. Additional analyses not presented in this manuscript are available to researchers from the corresponding author upon reasonable request.
