# Supplementary figures and images for: Cytokine dynamics and quality of life: unraveling the impact of cell-free and concentrated ascites reinfusion therapy in ovarian cancer patients
Source: Int J Clin Oncol. 2025 Jan 4;30(3):559–69. doi: 10.1007/s10147-024-02682-1 (PMC11842470; doi:10.1007/s10147-024-02682-1)

## Slide 1
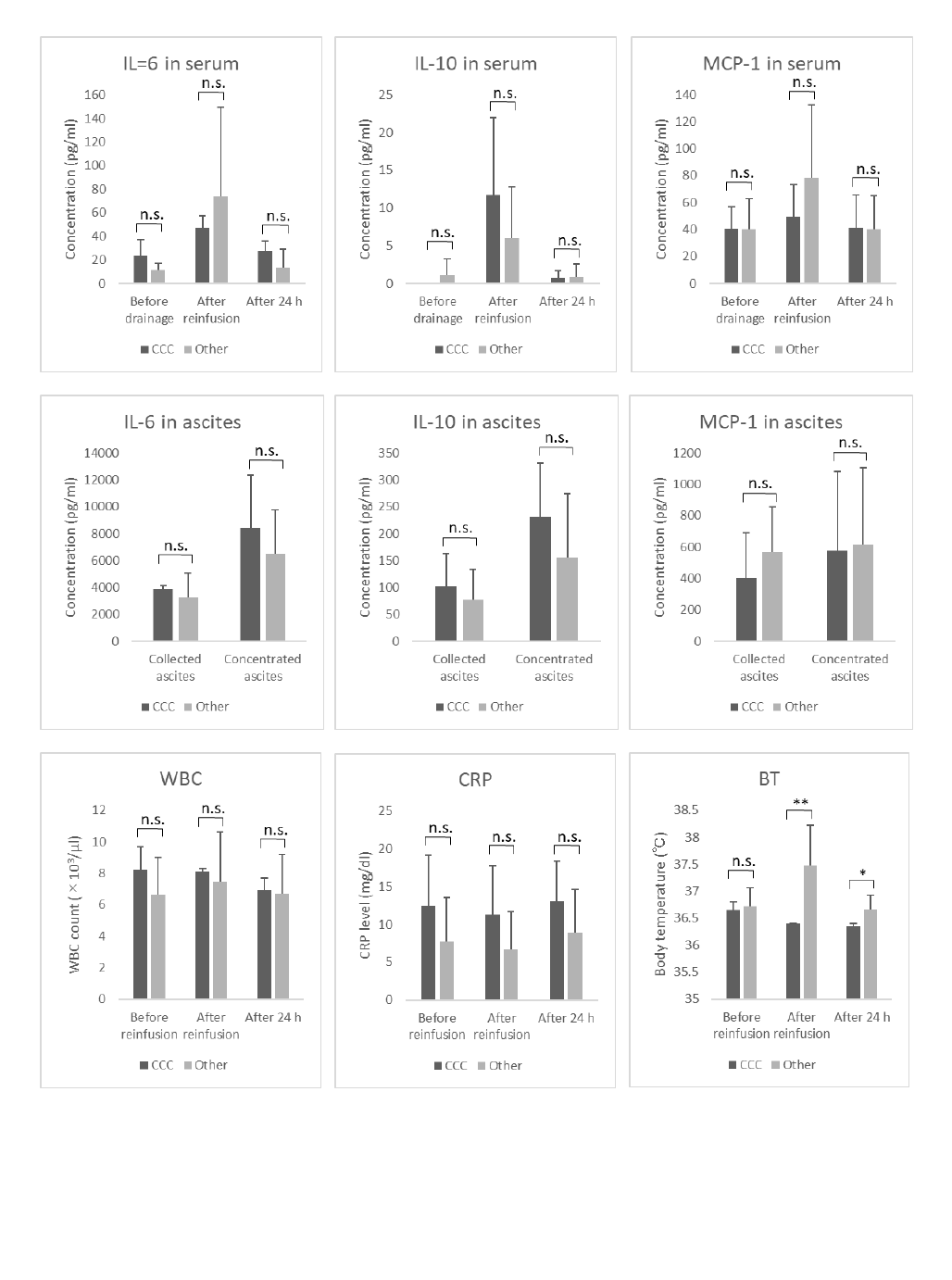

## Slide 2
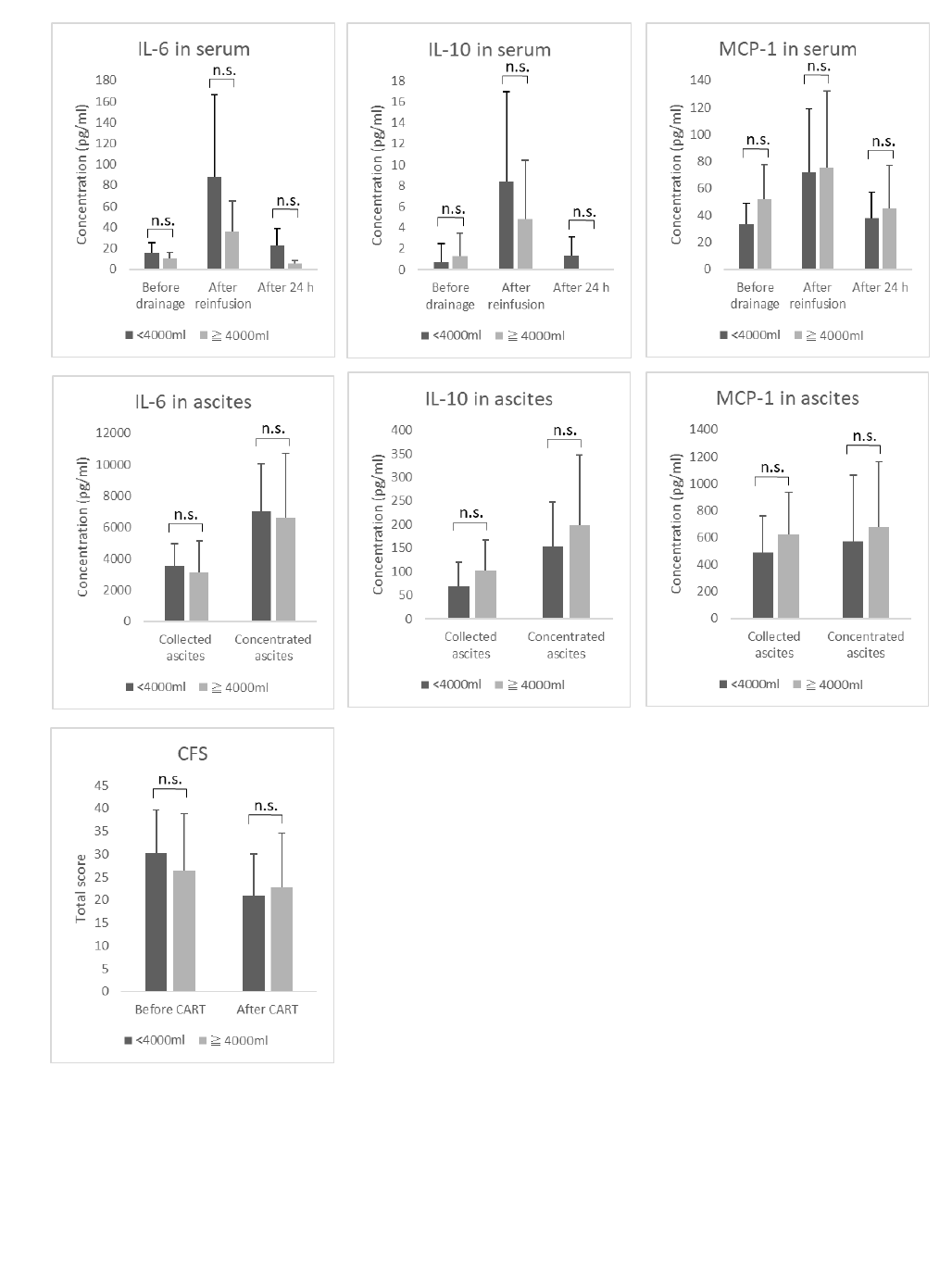

Supplement: Supplementary file 1 — Supplementary file1 (PPTX 150 kb) [file 10147_2024_2682_MOESM1_ESM.pptx]
