# Supplementary material for: Cytokine dynamics and quality of life: unraveling the impact of cell-free and concentrated ascites reinfusion therapy in ovarian cancer patients
Source: Int J Clin Oncol. 2025 Jan 4;30(3):559–69. doi: 10.1007/s10147-024-02682-1 (PMC11842470; doi:10.1007/s10147-024-02682-1)
Supplement: Supplementary file 2 — Supplementary file2 (DOCX 16 kb) [file 10147_2024_2682_MOESM2_ESM.docx]

| Serum | <4000ml | ≧4000ml | p value |
| --- | --- | --- | --- |
| IL-6 before drainage | 15.45 | 10.72 | 0.390 |
| IL-6 after reinfusion | 87.86 | 36.04 | 0.186 |
| IL-6 after 24 hours | 22.19 | 5.19 | 0.046 |
| IL-10 before drainage | 0.72 | 1.27 | 0.725 |
| IL-10 after reinfusion | 8.36 | 4.80 | 0.478 |
| IL-10 after 24 hours | 1.37 | 0.00 | 0.103 |
| MCP-1 before drainage | 33.28 | 52.29 | 0.299 |
| MCP-1 after reinfusion | 72.06 | 75.38 | 0.934 |
| MCP-1 after 24 hours | 37.96 | 45.29 | 0.729 |

| Ascites | <4000ml | ≧4000ml | p value |
| --- | --- | --- | --- |
| IL-6 collected ascites | 3548.95 | 3106.82 | 0.748 |
| IL-6 concentrated ascites | 7002.41 | 6576.85 | 0.881 |
| IL-10 collected ascites | 70.08 | 102.07 | 0.486 |
| IL-10 concentrated ascites | 153.45 | 198.74 | 0.652 |
| MCP-1 collected ascites | 490.35 | 619.74 | 0.571 |
| MCP-1 concentrated ascites | 571.66 | 676.96 | 0.770 |

| CFS | <4000ml | ≧4000ml | p value |
| --- | --- | --- | --- |
| Before CART | 30.14 | 26.33 | 0.720 |
| After CART | 21.00 | 22.67 | 0.868 |

The amount of ascites collected was divided into two groups: 4000 ml or more and less than 4000 ml, and the cytokines and QOL were compared.

The cytokine values and CFS are average values.

P-values were calculated by the student t-test.

CFS: Cancer Fatigue Scale
