# Supplementary material for: Cytokine dynamics and quality of life: unraveling the impact of cell-free and concentrated ascites reinfusion therapy in ovarian cancer patients
Source: Int J Clin Oncol. 2025 Jan 4;30(3):559–69. doi: 10.1007/s10147-024-02682-1 (PMC11842470; doi:10.1007/s10147-024-02682-1)
Supplement: Supplementary file 3 — Supplementary file3 (DOCX 17 kb) [file 10147_2024_2682_MOESM3_ESM.docx]

| Serum | CCC | Other | p value |
| --- | --- | --- | --- |
| IL-6 before drainage | 23.71 | 11.51 | 0.529 |
| IL-6 after reinfusion | 47.16 | 73.88 | 0.376 |
| IL-6 after 24 hours | 27.80 | 13.39 | 0.271 |
| IL-10 before drainage | 0.00 | 1.13 | 0.169 |
| IL-10 after reinfusion | 11.74 | 6.03 | 0.677 |
| IL-10 after 24 hours | 0.85 | 0.88 | 0.979 |
| MCP-1 before drainage | 40.57 | 40.11 | 0.983 |
| MCP-1 after reinfusion | 49.54 | 78.54 | 0.420 |
| MCP-1 after 24 hours | 41.53 | 40.42 | 0.971 |

| Ascites | CCC | Other | p value |
| --- | --- | --- | --- |
| IL-6 collected ascites | 3882.89 | 3278.24 | 0.401 |
| IL-6 concentrated ascites | 8458.19 | 6489.76 | 0.703 |
| IL-10 collected ascites | 102.31 | 77.13 | 0.749 |
| IL-10 concentrated ascites | 231.36 | 156.26 | 0.584 |
| MCP-1 collected ascites | 402.00 | 567.49 | 0.668 |
| MCP-1 concentrated ascites | 576.35 | 617.42 | 0.949 |

|  | CCC | Other | p value |
| --- | --- | --- | --- |
| WBC before reinfusion | 8.20 | 6.64 | 0.475 |
| WBC after reinfusion | 8.10 | 7.47 | 0.589 |
| WBC after 24 hours | 6.95 | 6.72 | 0.849 |
| CRP before reinfusion | 12.51 | 7.74 | 0.600 |
| CRP after reinfusion | 11.33 | 6.67 | 0.602 |
| CRP after 24 hours | 13.11 | 8.92 | 0.565 |
| BT before reinfusion | 36.65 | 36.72 | 0.737 |
| BT after reinfusion | 36.40 | 37.48 | 0.003 |
| BT after 24 hours | 36.35 | 36.67 | 0.016 |

Comparison between clear cell carcinoma and other histologies.

Values for cytokines, WBC, CRP, and BT are averages.

P-values were calculated by the student t-test.

CCC: Clear Cell Carcinoma, WBC: White Blood Cell, CRP:C-Reactive Protein
