# Supplementary material for: Cytokine dynamics and quality of life: unraveling the impact of cell-free and concentrated ascites reinfusion therapy in ovarian cancer patients
Source: Int J Clin Oncol. 2025 Jan 4;30(3):559–69. doi: 10.1007/s10147-024-02682-1 (PMC11842470; doi:10.1007/s10147-024-02682-1)
Supplement: Supplementary file 4 — Supplementary file4 (DOCX 15 kb) [file 10147_2024_2682_MOESM4_ESM.docx]

| IL-6 | equation | R^2^ | p value |
| --- | --- | --- | --- |
| Age | y=0.8961x-5.0723 | 0.01097 | 0.759 |

| CFS | equation | R^2^ | p value |
| --- | --- | --- | --- |
| Age | y=-0.1146x15.2145 | 0.04815 | 0.542 |

The single regression analysis conducted to evaluate the relationship between IL-6 levels and CFS (Clinical Frailty Scale) scores by age
